# Effects of the CarFreeMe Traumatic Injuries, a Community Mobility Group Intervention, to Increase Community Participation for People With Traumatic Injuries: A Randomized Controlled Trial With Crossover

**DOI:** 10.3389/fneur.2022.821195

**Published:** 2022-07-01

**Authors:** Stacey George, Christopher Barr, Angela Berndt, Rachel Milte, Amy Nussio, Zoe Adey-Wakeling, Jacki Liddle

**Affiliations:** ^1^Caring Futures Institute, College of Nursing and Health Sciences, Flinders University, Adelaide, SA, Australia; ^2^Department of Occupational Therapy, College of Nursing and Health Sciences, Flinders University, Adelaide, SA, Australia; ^3^Department of Rehabilitation, Aged and Palliative Care Services, Flinders Medical Centre, Bedford Park, SA, Australia; ^4^Department of Occupational Therapy, Allied Health and Human Performance, University of South Australia, Adelaide, SA, Australia; ^5^Department of Rehabilitation and Extended Care, College of Medicine and Public Health, Flinders University, Adelaide, SA, Australia; ^6^School of Information Technology and Electrical Engineering, The University of Queensland, Brisbane, QLD, Australia

**Keywords:** trauma injuries, traumatic brain injury, spinal cord injuries, group-based intervention, community mobility, participation, community participation

## Abstract

**Introduction:**

After traumatic injuries community participation is a common goal, promoting wellbeing and independence. Community mobility and transportation influence an individual's independence in community participation. With the ability to drive safely often compromised after traumatic injuries, the adverse consequences of driving cessation include a loss of identity and reduced participation in chosen activities. In rehabilitation, individualized community mobility intervention is not routinely provided. The primary aim of this trial was to evaluate whether a group-based intervention, the CarFreeMe TI program was more effective than standard intervention, an information sheet of alternative transport, in improving community mobility for people following traumatic injuries. The secondary aim of this study was to evaluate the effect: types of transport used, transport satisfaction, community mobility self-efficacy, quality of life, goal satisfaction and performance, for people following traumatic injuries; and to undertake a preliminary assessment of the potential resource use associated with the intervention, and lessons for implementation.

**Design:**

Prospective, pilot, randomized, blind observer, controlled trial with crossover.

**Participants:**

Twenty individuals with traumatic injuries.

**Intervention:**

Six-week group-based support and education program, the CarFreeMe TI delivered in community settings (intervention) and standard information related to transport options available (control).

**Primary Outcome Measures:**

Community participation using a Global Positioning System device to record the location and number of outings from home.

**Secondary Outcome Measures:**

CarFreeMe TI Transport Questionnaire, Community Mobility Self-efficacy Scale, quality of life measures, Modified Canadian Occupational Performance Measure for goals (importance and satisfaction), participant satisfaction survey results and researcher logs.

**Results:**

Those who received the intervention were more likely to use public transport and transport services and had an improved quality of life, when compared to the control group. The intervention group also reported high levels of improvement in goal performance and satisfaction. Global Positioning System data collection was incomplete, with geolocation data unusable. There was no significant change in number/type of visits away from home.

**Conclusions:**

A group-based community mobility education program promoted modes of active independent transport but did not impact on outings from home. Future research could include passive collection methods using a smartphone to record community participation.

**Clinical Trial Registration:**

https://www.anzctr.org.au/, identifier: ACTRN12616001254482.

## Introduction

Injuries caused through trauma, including traumatic brain injuries (TBI) and spinal cord injuries (SCI) are a leading cause of disability (1, 2), internationally. In rehabilitation, community participation is a common goal for people with traumatic injuries (TI), aiding engagement in meaningful and chosen activities (3, 4). Community participation can be considered within the International Classification of Functioning and Disability (ICF) (5). In the ICF framework: Activities are the execution of a task or action by an individual; Participation is the performance of people in activities across social life domains, through interaction with others; and community participation is the performance in activities across the domains of: (1) domestic life; (2) interpersonal life (entailing formal and informal social, family and intimate relationships); (3) major life activities including education (informal, vocational training and higher education) and employment (remunerative and non-remunerative, excluding domestic work); and (4) community, civic and social life (including religion, politics, recreation and leisure, hobbies, socializing, sports, arts and culture); of an individual in the context of the community in which they live.

Success of community participation is markedly influenced by community mobility and transportation, enabling access to healthcare services, independence and participation in daily activities (6–8). Following TBI and SCI, driving, a form of transportation, has been identified as a key activity to maximizing community participation (7, 9). Generally driving is the most accessible and highly valued transport option, particularly so for the generally younger and male demographic of people acquiring a TBI or SCI injury in developed countries (10).

However, the capacity to drive safely can be compromised after traumatic injuries, as driving is a complex task involving a high level of physical, sensory, perceptual and cognitive functions integrated in an unpredictable and challenging environment. This reduced safety in driving for individuals after traumatic injuries, can be related to changes in physical functions, visual abilities (11), judgment and attention in TBI (12), and sensory awareness and muscle strength in SCI. Research indicates that a proportion of people return to driving at some stage post-traumatic injury, with rates of between 36 (13)−50% (14) following TBI and 36.5% (7) following SCI. Thus, at least half of the population who sustain severe TBI and 63% of people following SCI are unable to return to driving.

Driving cessation after having a complex traumatic injury is associated with emotional, identity, transport and participation related needs (9), and leads to a reduced quality of life for the individual (9). Furthermore, following a traumatic injury, such as TBI and SCI, much adjustment to returning to valued life roles is required by the individual (15). As such the actual adjustment to driving cessation has been found to be an important component to successful community participation, and a unique and continuing experience for individuals with traumatic injuries (9).

Returning to valued life roles, like being a driver, are important for life satisfaction after injuries, such as TBI and SCI (16). Research is required to examine participation in life roles, after traumatic injuries, specifically the personal importance and changes in these life roles (including driving and community mobility). Additionally, it is critically important to develop education and intervention programs to address these specific life roles to maximize life satisfaction after traumatic injuries (16). Research demonstrates that intervention to facilitate community mobility in the context of driving cessation following traumatic injuries needs to target the emotional, social and practical issues in a personalized way (9), specifically in relation to the life role of driving and community mobility.

Following traumatic injuries, including TBI and SCI, community rehabilitation in developing countries is often provided through government supported insurance schemes. Presently, individualized community mobility intervention is not routinely provided in these rehabilitation services (17). Rehabilitation programs in developed countries offer community mobility interventions comprising of: driving assessment, information describing alternative forms of transport options, and the coordination of multidisciplinary support (9, 17). With successful intervention to maximize community mobility for people following traumatic injuries, being required to be individualized, ongoing and include: 1. information provision; 2. support to facilitate adjustment and decision-making; 3. goal setting and; 4. practical support to use alternative transport; in order to maximize participation in valued roles. Explorations of the experiences of key stakeholders, has identified that the needs related to driving cessation were affected by the processes of formal driving requirements, rehabilitation, adjustment and support available (9). A flexible, individualized approach that considered emotional and practical needs was indicated (9).

The CarFreeMe intervention (http://carfreeme.com.au/) is a community-based education and support program, developed in Queensland, Australia which has been demonstrated in a randomized controlled trial to increase community mobility and transport satisfaction in older adults following driving cessation (18). CarFreeMe TI, where the TI refers to Traumatic Injuries (TI), a modified version of the program, thereby enhancing the ecological validity, that is the real world application to the specific needs of people who are unable to drive, resulting from traumatic injuries, was developed through expert clinical input and a review of relevant research. Modifications included introduction of an optional family module, adaptation of language, examples and images to ensure relevance to this group, and inclusion of traumatic injury specific content (including licensing/fitness to drive requirements, impact of symptoms on driving and alternative transport use, and reference to rehabilitation pathways) and specific traumatic injury related organizations/resources. Modifications to the program were made in consultation with experienced clinicians, researchers, service providers, and advocacy organizations in the area.

The CarFreeMe TI intervention program consists of seven modules run over 6 weeks, with sessions on: adjusting to losses and changes (including mindfulness and relaxation techniques, cognitive behavior therapy approaches); experiences of stopping driving; alternative transport; lifestyle planning (how to stay involved without driving, planning for the future); and advocacy and support. An additional family caregiver module was added in recognition of the important and challenging role of family members of people after traumatic injuries in driving cessation support. Group activities took the form of information sharing, group discussion, speakers, practical sessions and outings facilitated by an occupational therapist and a peer leader (a person living with a TBI or SCI and no longer driving, mentored by the occupational therapist). The approach and rationale to engaging peer leaders in the driving cessation program has been described elsewhere (19). The order and focus within modules were also adapted depending on individual goals, which are set before the first session. For example, if group members had priority goals on finding alternative transport, or advocating for change within the local area, the relevant modules (numbers 5 and 7) would form part of early sessions. In addition, the nature of goals (e.g., organizing transport to a future study location, to feel okay discussing not driving) were reflected in planned guest speakers, outings and worked examples. Individual sessions, homework and individual transport plans also reflected individual goals. Table 1 contains an overview of the modules and example content and activities. In terms of implementation of the program, the factors considered included the length of sessions and frequency of breaks to consider mental fatigue, and individual sessions for practical training in actual contexts (i.e., catching the bus from own home to University or gym), in addition to a group based outing that provided more generalized exposure to public transport use.

**Table 1:** Modules and example content and activities of the CarFreeMe-TI program.

**Module**	**Example content and activities**
1. Traumatic injury	Nature of experiences and changes Challenging myths, perceptions of those in society Activities: Discussing experiences, perceptions; Guest speaker from related services;
2. Balancing safety and independence	Formal requirements and processes for licensing Impact of symptoms on driving safety Activities: Presentation on local requirements and driving assessment experiences Discussion (including unlicensed driving)
3. Adjusting to losses and changes	Grief, loss and coping styles Activities: Worked examples (cognitive behavioral/challenging thinking; problem solving) Relaxation exercises-Meditation guest speaker
4. Experiences of giving up driving	Sharing experiences, expectations, concerns about driving cessation Activities: guest speaker (peer leader or other), sharing experiences, discussion, workbook activities
5. Alternative transport	Information about local options (ticketing, concessions, getting information, planning trips) Practical experience with relevant options Activities: group and individual outings and reflection; Individual Transport Plan, Guest speaker from public transport service, Peer support in a targeted guest speaker role in relation to using alternate transport
6. Lifestyle planning	Reflecting on personally optimal lifestyle Occupational balance, pacing/energy conservation Activities: workbook led reflection on current time use patterns, reflection and discussion of goals/ future planning
7. Advocacy and support	What is advocacy Current and future opportunities for transport, support Activities: Supported feedback of local area audit, connection with advocacy groups; Guest speaker: local council member
8. Family member (optional)	Conversations during driving disruption Support for carers/family members Activities: Discussion about experiences, Guest speaker from carer support organization or peer

Currently no intervention programs, specifically targeting community mobility such as CarFreeMe TI regarding driving cessation and community mobility are available for people with complex traumatic injuries in Australia within standard practice. There are high costs to the community for providing rehabilitation, community support (20, 21), and also non-travel and non-participation to people following TBI and SCI. Moreover, there are no published studies evaluating the clinical effectiveness or efficiency in terms of cost effectiveness of interventions targeting enhanced community mobility in people following traumatic injuries (22, 23). There are also relatively few economic evaluations of rehabilitation strategies following severe traumatic injuries (24–27), with those that are published predominantly focusing on cost-analysis or cost-benefit analysis.

When considering targeting intervention at community participation, the use of outcome measurements to evaluate effectiveness needs to be considered. A recent scoping review recommends a mixed method approach including Global Positioning Systems (GPS) for quantitative data (distance and location), and qualitative data including self-reported participation diaries to provide insight into where and why individuals chose to complete certain community activities (28). GPS has been used effectively in older community dwelling adults (29) and people with lower limb amputations (30) to measure community participation in observational cross-sectional studies. Other research investigating community participation uses the construct of lifespace, the geographic area where an individual lives and carries out their life. Lifespace measurement has been used mostly in relation to older people including those with mild cognitive impairment and dementia (31) and is measured more recently through the passive collection of the recording of outdoor locations using GPS on a smartphone. The data are then converted to metrics including areas, percentage of time at home and number of times leaving the home.

The primary aim of this trial was to establish whether a 6-week CarFreeMe TI intervention focused on improving community mobility is more effective than standard intervention, in people following traumatic injuries, vs. a standard intervention on: types of transport used, transport satisfaction, community mobility self-efficacy, quality of life, goal performance and satisfaction, participant satisfaction, for people following traumatic injuries; Carer's self-efficacy and strain; and to undertake a preliminary assessment of the potential resource use associated with the intervention, and lessons for implementation.

## Materials and Methods

### Study Design

This study was a prospective, randomized, blind observer, controlled trial with crossover, following the guidelines of the Consolidated Standards of Reporting Trials (32). The protocol has previously been published (33).

### Participants

**Figure 1:**
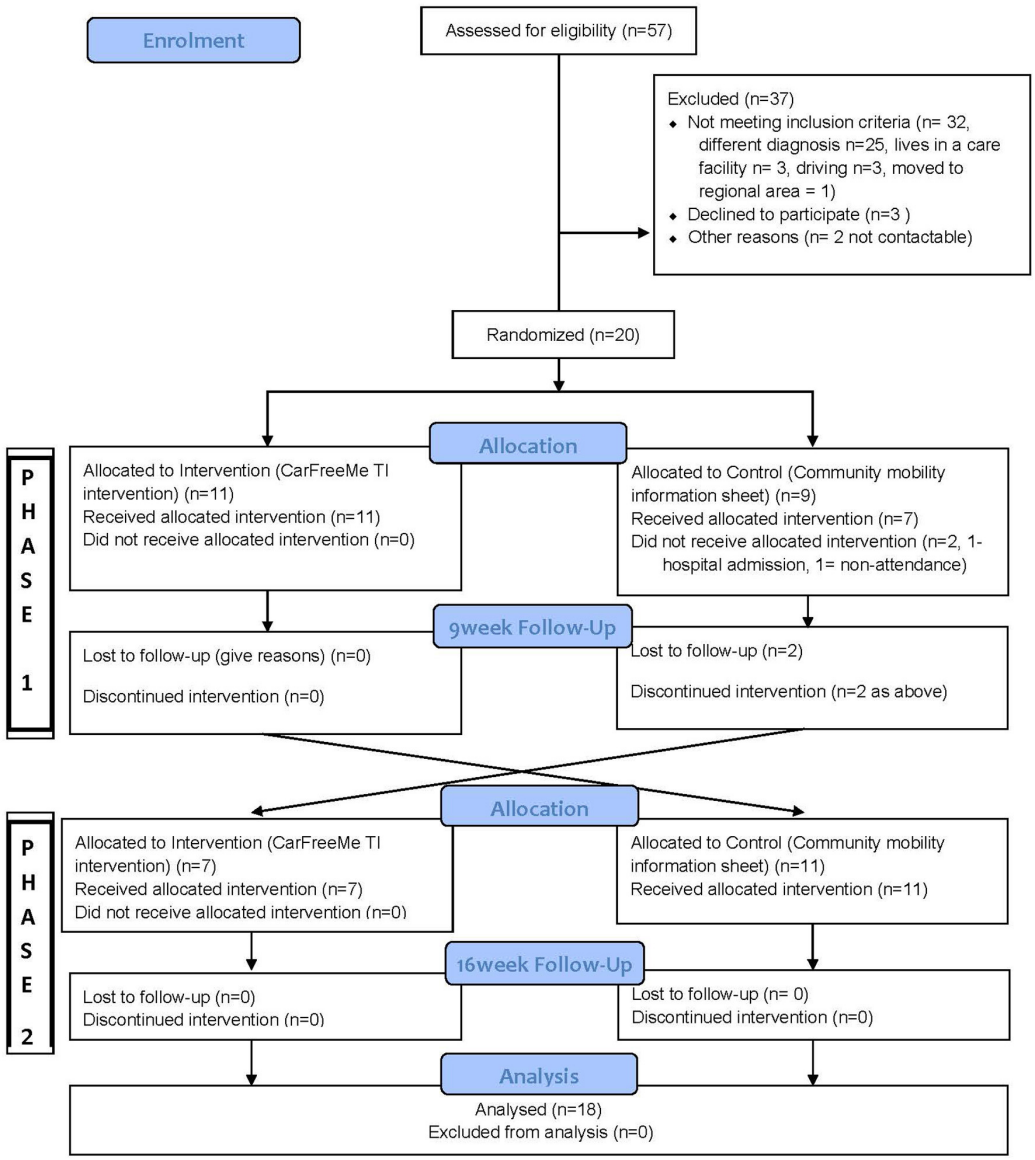
Flow diagram.

Fifty seven individuals with traumatic injuries recruited from rehabilitation facilities and the community, in Adelaide South Australia, were screened for eligibility between July 10, 2016 and July 25, 2017 by a research Occupational Therapist. Of these, 32 were ineligible, with reasons detailed in the Flow Diagram (Figure 1) of those who were deemed eligible three declined and two were not contactable. Twenty participants were included in the study. The inclusion criteria were as follows: (a) had a traumatic injury, that is a TBI and/or a SCI, which precludes returning to full driving; (b) aged over 18 years old; (c) adequate cognition/behavioral abilities to participate in sessions; (d) mobile, either walking or in a wheelchair independently or with carer assistance. The research assistant who made the assessment of suitability to participate, was an Occupational Therapist with experience working with people with traumatic brain injury, and their clinical judgment, and knowledge of the CarFreeMe program and options for adaptation, and information from referees which included Occupational Therapists providing intervention, informed the assessment of suitability to participate. If there was concern about the participant's ability to make decisions, capacity to consent was confirmed from the treating doctor, with permission from the client. Exclusion criteria were as follows: (a) evidence of aphasia or poor English language skills that significantly impact on the understanding of information and reduces engagement in a group setting; (b) living in residential care settings (or anywhere where transport would be provided); (c) driving with no restrictions.

Participants were recruited from urban areas in Adelaide, South Australia. The urban area is sprawling, with public transport predominantly being buses, with limited options for trains/trams. Supported transportation services are available for people with disabilities from local councils and community service providers for activities such as shopping or medical appointments. Driving a motor vehicle is the predominant form of transport and national medical fitness to drive guidelines https://austroads.com.au/publications/assessing-fitness-to-drive inform decision-making related to driving for people with traumatic injuries.

The study was approved by the ethics committee of Southern Adelaide Clinical Health (OFR # 42.16 – HREC/16/SAC/47) and all participants provided written informed consent. All procedures were conducted in accordance with the Helsinki Declaration of 1975, as revised in 2008. The study was registered with the Australian and New Zealand Clinical Trials Register (https://www.anzctr.org.au/ACTRN12616001254482).

### Procedure

In Phase One of the study, participants underwent a baseline assessment and were randomly allocated to either 6 weeks of the CarFreeMe TI support and education program (intervention) or received information related to transport options (control). In Phase Two of the study those who were in the intervention group crossed over to receive the control and vice versa. A computer-generated randomization schedule with one:one allocation occurred by an investigator not involved in recruitment or assessments.

Primary and secondary outcome assessments were performed at baseline, before any interventions (Week One-Two), Week Nine (following completion of Phase One) and week 16 (following completion of Phase Two). Assessors, blinded to the group allocation, were research Occupational Therapists who have received training in the standardized use of the outcome tools.

### Instruments

#### Primary Outcome

The primary outcome measure of community participation was a Global Positioning System (GPS) to record the location and number of outings from home. Hordacre et al. (30) and Gough (29) were able to accurately record a range of categorized community participation events using wearable GPS devices integrated with geographic information systems. The GPS units were worn for a 7-day period at the end of the 6-week intervention, at each phase of the study. Participants were provided with a GPS device, as used in our previous studies in amputees (30) and older community dwelling adults (29). The device was worn on a lanyard or belt hook for a period of seven consecutive days and shows location during daily community activities. The particular GPS model used was the QSTARZ BT-Q1000XT, considered the gold standard for research (29). The device measures 72.2 mm (L) × 46.5 mm (W) × 20.0 mm (H), weighs eight and a half grams. Battery life of the device was 42 h, and participants were instructed to charge the device each night. The Occupational Therapist regularly checked in with participants via telephone to remind participants to wear the tracker and instructions/education were provided to participants and carers. Researchers were unable to check the data remotely to check if the GPS was accurately recording. The data collected provided longitude and latitude coordinates time-stamped for every 5 s. Data were linked to Google Maps for a graphic representation of where participants traveled within the community. Participants were made aware of the nature of the data that is obtained from the GPS device prior to giving consent to participate in the study.

From the coordinate data, the following were calculated: Trips per day, furthest distance traveled, average daily distance and percentage of time at home. The types of places participants visited were categorized such as: employment, residential, commercial, health services, recreational and social.

#### Secondary Outcomes

The secondary outcomes measured offer a broader picture of quality of life, health care resource use, transport use, and confidence with participation without driving, for participants and confidence and strain for carer's and included:

- **CarFree Me TI Transport Questionnaire:** included data on modes of transport used, as well as a diary record of community mobility in the last 7 days to support GPS data. Basic satisfaction with transport arrangements was measured by a five-point scale with five very satisfied and one very dissatisfied. This questionnaire was developed for the study evaluating the effectiveness of the CarFreeMe for older drivers (19) which found the intervention was significantly associated with a higher number of episodes away from home per week and an increase in modes of transport and higher satisfaction with transport use. Psychometric properties of this bespoke questionnaire are not available. In the original CarFreeMe trial, a difference of one additional trip in the community within a week was defined as clinically meaningful (19).
- **Community Mobility Self-efficacy Scale:** measured participant confidence with participation in life roles and activities without driving. For example, “How confident do you feel about being able to stay in contact with friends and family without driving?” This questionnaire assessed the level of confidence on a ten-point scale ranging from one, not confident at all, to ten, very confident. This was developed from an adaptation of Lorig et al. (34) scale for a study evaluating the effectiveness of the CarFreeMe intervention with older drivers (19). The results of this study were that some features of the Community Mobility Self-efficacy Scale demonstrated significant improvements following intervention including: “How confident do you feel about being able to stay involved in the community without driving?”, Item five: “How confident do you feel about finding alternative transport options to get to necessary community activities and appointments?”, and Item six: “How confident do you feel about staying involved in activities that are important to you without driving?” (19). Psychometric properties for this adapted scale are not available. The scale from which it was developed has indicated high internal consistency, sensitivity to change and appropriate correlation with relevant health outcomes in the context of chronic disease self-management programs, over a range of contexts and languages (35). There has not been a formal indication of clinically significant magnitude of change in this scale of which we are aware.
- **Health-related quality of life of participants** was measured using two instruments: the Assessment of Quality of Life Six Dimension (AQoL-6D) (36) and EuroQoL 5 dimensions five levels (EQ-5D-5L) (37). The AQoL-6D is an instrument which measures health-related quality of life across six dimensions, independent living, mental health, coping, relationships, pain, senses, and visual impairment. There are 20 questions in total for the instrument. Participants were asked to rate their situation over the previous week. The AQoL-6D can be scored a number of ways, including using a simple additive summary score to give an indication of overall quality of life, where scores range from 20 to 97 where a lower score indicates a better quality of life (38). The instrument has the required validity (construct, concurrent, and convergent) (39), has undergone psychometric construct and validation processes and generates health utilities that are comparable with other major health utility instruments (40). The EQ-5D-5L is a generic-preference based instrument for measuring health-related quality of life which has five questions covering five dimensions (mobility, self-care, usual activities, pain and anxiety and depression) plus a visual analog scale (VAS) which asks participants to rate their overall health on a zero (indicating the worst health imaginable) to 100 (the best health imaginable) scale. It is described as having excellent psychometric characteristics across setting and groups, having moderate responsiveness in groups experiencing health improvements (41). Participants are asked to rate how they would describe themselves across the five questions today using the five possible levels of response. The EQ-5D-5L can be used to generate utility scores which are scores indicating the overall quality of life weighted according to the preferences of the general population for the health state described by the five dimensions. The EQ-5D-5L was scored using the weighted scoring algorithm generated from the preferences of the Australian general population, which gives scores on a scale between zero and one, where zero indicates a health state equivalent to death, and one the best possible health state (42).
- **Individual goals:** were set only by participants undertaking the intervention condition, just prior to the group starting. Participants were assisted to set transport and lifestyle goals using a modified Canadian Occupational Performance Measure (COPM) (43). The COPM has been described as clinically useful, responsive, valid and reliable (44). Participants identified their goals for participation and rated their current performance, and satisfaction for each goal on a 10- point Likert scale ranked from one-10, where one indicated poor performance and low satisfaction, respectively, whilst 10 indicates very good performance and high satisfaction. Goal performance and satisfaction were rerated at the completion of the intervention. This is consistent with the approach undertaken in the trial with older people (45). Clinically meaningful change in the COPM has been defined as a change of two or more points (46).
- **Cost and resource use**: A health-system perspective was adopted for the analysis of the costs and resource use within the study. The costs of the intervention were calculated using study based records of the Occupational Therapists time, and resources used. Health and aged care service utilization was accessed from self-reported weekly records of service use. Participants were asked to complete a calendar recording instances of health and social care services input such as care provided at home, visits from allied health professionals or to clinicians. Unit costs for the health system resources used to provide the intervention were derived from health service data. Costs for the other resources used in the intervention were based on market rates.
- **Participant satisfaction survey and researcher logs:** A satisfaction questionnaire was completed at the end of the group education and included questions rated on a Likert scale of one-10, where one indicated not satisfied and 10 extremely satisfied related to content, presentation and organization of the education program. This represented a bespoke questionnaire where psychometric properties are not known. Then open questions related to what you would keep, take out, relevance, influence on knowledge/confidence and suggestions for improvment.The Occupational Therapist research assistant also maintained logs throughout the study.
- **Carers outcomes:** For the carers of participants with traumatic injuries who consented to participating in the trial, including the option of also attending the CarFreeMe TI group sessions, outcomes included the Carer's Community Mobility Self-efficacy Scale and the Modified Carer Strain Index. The Carer's Community Mobility Self-efficacy Scale is a 10 point Likert scale measuring perceived confidence of participants ability maintaining community mobility following driving cessation adapted from the Community Mobility Self Efficacy Scale (18); The Modified Carer Strain Index (47) is a questionnaire of 13 items measuring perceived burden of carers rated on a Likert scale ranging from “experiencing on a regular basis, sometimes, to not at all”. This tool has been used with a range of caregivers, is brief and convenient and has high internal and test-retest reliability, and has been used across cultures and languages (47).

Adverse effects were monitored including unlicensed driving and any injuries related to community mobility, and referral to support services (physiotherapy, psychologist, social work) made if required, as determined by the research Occupational Therapist. A steering committee consisting of authors and representatives from Paraquad South Australia (SA), Brain Injury South Australia oversaw the monitoring of data and dissemination.

### Intervention

Both interventions were provided by an experienced rehabilitation Occupational Therapist (AN) who had received training by the developer of the CarFreeMe TI (JL) program.

### Intervention Protocol

The intervention was a group-based support and education program, the CarFreeMe TI delivered in community settings. Prior to commencement of the group a home visit was conducted to identify individual goals and discuss practical challenges with group attendance. The intervention included up to eight participants per group, with six sessions conducted once a week and each session was up to two and a half hours in duration. Content of sessions followed an established protocol and included: adjusting to loss and change; experiences of stopping driving; alternative transport; lifestyle planning; and advocacy and support. Session content was modified to be relevant to the goals identified by group members and were interactive and facilitated for information sharing, using peer leaders and guest speakers including meditation experts, representatives from local council and carer support organizations. Outings were also included which offered the opportunity to trial alternate transport methods such as public transport. Attendance was recorded at each session by the Occupational Therapist, to monitor adherence.

### Control Protocol

The control intervention received standard information related to transport options available, which was a one-page written information sheet.

### Data Analysis

Sample size was calculated based on data reported in a study assessing community participation in amputees (30). In this study, the mean number of community participation visits over the course of a continuous week for amputees classified as having limited mobility was 7.2, and for those with basic to normal activity was 13.7. Using the group SD of 10.9, and assuming a power of 0.8 and alpha of 0.05, a sample size of 45 would be required to detect a similar difference. Assuming a 20% drop-out, we therefore aimed to recruit 54 participants.

Data was entered into an SPSS database with all identifying information removed. Statistical analysis was undertaken using SPSS version 23 Statistical software (IBM, Chicago). Intention-to-treat analysis was conducted and was blinded (i.e., groups identified by number only).

Baseline demographics were compared between the groups using Mann-Whitney *U-*tests, as the data was not normally distributed, for continuous variables and *X*^2^ for categorical variables. For Phase One, at the end of the RCT section of the study, differences in community participation were assessed via GLM Analysis of Variance (ANOVA) with group allocation and time as factors. After Phase One was completed and no carry over effect was confirmed the data from Phase One and Phase Two were pooled to allow analysis as a pre-post study. Paired *t*-tests were conducted to assess the effect of the intervention on the use of transport methods for all participants. Finally, the frequency of episodes away from home for each category of location and total episodes recorded were compared for all participants between baseline, after the intervention period, and after the control period, using a repeated measures ANOVA. For all outcomes alpha was set at 0.05.

A preliminary exploratory study of the health-service resource use and costs associated with the intervention was undertaken. Costs accrued over the 6 week intervention period were estimated for each participant. Descriptive statistics were calculated for the costs of providing the intervention. The nature of the goals set prior to intervention participation were analyzed using an inductive content analysis (48). Goals were worded using the expression of participants. These were deidentified, read and grouped according to key content areas apparent in the data by a member of the investigator team (JL). Preliminary coding was checked and verified by other members of the research team (AN, SG). Feedback about the experience of group participation was analyzed in the same way.

## Results

Twenty participants with a mean age of 53.8 years (SD 13.9 years) were recruited to the study. Types of injuries included Traumatic Brain Injury–16, Spinal Cord Injury–3, and Orthopedic–1. Median time since injury was 6.0 years with Inter Quartile Range (IQR) 1.9–18.8. Participants in Group One and Group Two had no significant differences in demographics (see Table 2). A Mann-Whitney *U* Test revealed no significant difference in the age in Group One (Md = 56, *n* = 11) and Group Two (Md = 58, *n* = 9), *U* = 63.5, *z* = 0.29, *p* = 0.30, *r* = 0.0.06, and time since injury (months) in Group One (Md = 97, *n* =11) and Group Two (Md = 46, *n* = 9) *U* = 43, *z* = −0.50, *p*= 0.66, *r* = 0.11. A Chi square test revealed no significant differences between Group One and Group Two in gender [X^2^_(1)_ = 1.63, *p* = 0.20], type of injury [X^2^_(3)_ = 1.81, *p* = 0.6] and referral source [X^2^_(3)_ = 1.81, *p* = 0.61]. Two participants in the control group withdrew, one due to a hospital admission and one due to non-attendance at subsequent outcome assessment timepoints. Table 2 shows a baseline comparison of demographics.

**Table 2:** Baseline comparison of demographics between groups.

	**Group 1 (*n* = 11)**	**Group 2 (*n* = 9)**	**Statistic**	***p*** **-value**
Age years median (IQR)	56 (35–59)	58 (53–64.5)	*U* = 63.5	*0.30*
Age years range	21–69	46–80		
Male *n* (%)	9 (82)	5 (56)	*X*^2^ = 1.63	*0.20*
Time (months) since injury Median (IQR)	97 (25–209)	46 (15–330)	*U = 43*	*0.66*
Time months since injury range	10–548	10–507		
Injury type			*X*^2^ = 1.81	*0.61*
Traumatic Brain Injury (TBI)	10 (91%)	6 (67%)		
Spinal Cord Injury (SCI)	0	2 (22%)		
TBI/SCI	0	1 (11%)		
Orthopedic Injury	1 (9%)	0		
Referral source			*X*^2^ = 4.23	*0.12*
Self	2 (18%)	4 (44%)		
Lifetime Support Agency (Public Insurer)	1 (9%)	1 (11%)		
Inpatient rehab	1 (9%)	1 (11%)		
Community rehabilitation	9 (%)	3 (%)		

### Primary Outcome

In terms of the primary outcome measure, the Global Positioning System device, average data collection was low (mean 8.3–17.6%) and thus could not be included in the analysis. In the other studies (28, 29) 80–90% has been used as the cut point for “complete” data. In this study the mean of eight-17 across the three timepoints is artificially high because at each point one full data set (over 90%) was there for one person, with the rest of participants averaging 1–2%. No individual had a full set of data—three individuals had a full set of data at one timepoint only. Reasons reported by the Occupational Therapist research assistant describes participants turned the devices off when they were at home, they did not turn them on properly when turning them back on, they pressed a button that changed the data collection mode, did not charge nor take the devices with them when left the house.

**Figure 2:**
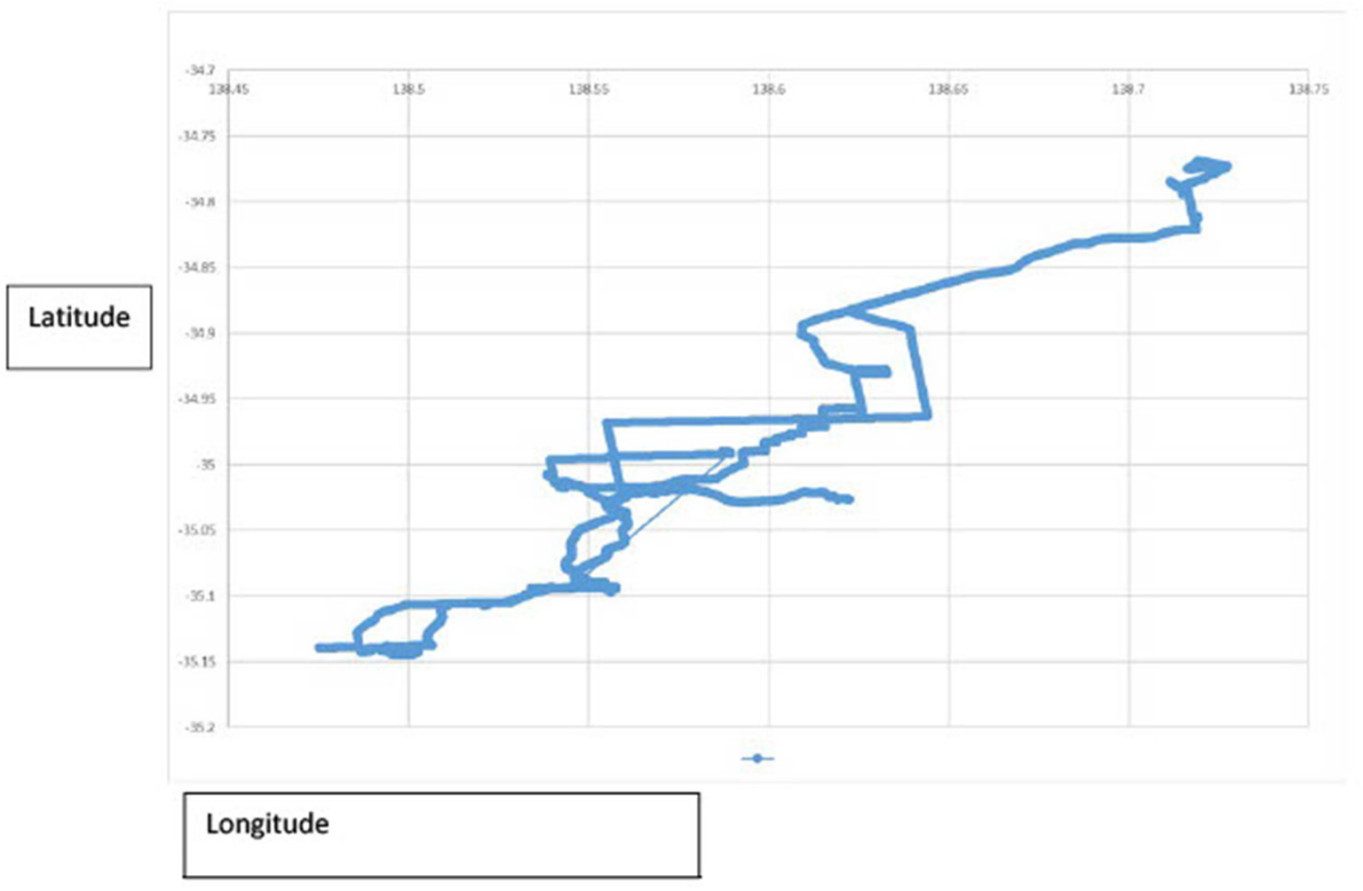
Global positioning system data.

A pictorial presentation of the GPS data from one participant is presented in Figure 2—this can be overlaid onto a map to identify location, however was not for publication purposes for deidentification, demonstrates for this participant that using public transport they traveled 60 km in one outing from home to attend study activities.

### Secondary Outcomes

#### CarFree Me TI Transport Questionnaire

The total number of trips out of home and the use of different transport methods used at the end of phase one of the study are presented in Table 3. There was a significant effect of group allocation over time for the use of a transport service, indicating an increase in service use in the intervention group (*F* = 5.102, *p* = 0.037). Supported transport services included the equivalent of transport provided by disability services, local government council drivers and courtesy buses, that are required to be organized in advance of a trip. Despite the increase in use of a service for the intervention group over time there was no significant interaction of group allocation over time for any of the other methods or total trips out of home (*F* = 1.093, *p* = 0.310).

**Table 3:** Use of transport methods to leave the home M (SD) over a 7-day period at the end of Phase 1.

	**Baseline**		**9 week**	
**Type of transport**	**Intervention**	**Control**	**Intervention**	**Control**
Walking	2.7 (4.4)	0.4 (1.1)	1.3 (3.6)	0.6 (1.2)
Bus	2.2 (3.0)	1.9 (4.2)	3.5 (4.3)	2.0 (3.7)
Train	0.7 (2.4)	0.9 (2.5)	0.7 (1.6)	0.9 (2.5)
Taxi	0.5 (0.8)	1.3 (1.9)	1.6 (1.9)	1.4 (2.7)
Service^*^	1.0 (3.3)	0.4 (0.7)	1.4 (3.6)	0 (0)
Lift	4.7 (3.6)	4.1 (3.2)	2.8 (4.0)	4.8 (5.1)
Courtesy bus	0 (0)	0.1 (0.4)	0.1 (0.3)	0 (0)
Other	1.6 (2.5)	1.2 (2.8)	1.7 (2.1)	1.8 (4.9)
Total	7.3 (3.6)	5.9 (2.3)	5.6 (2.2)	6.0 (4.1)

* *Significant interaction effect of group allocation by time*.

As there was no effect of group allocation on any aspect of transport uses, the data was pooled and treated as a delayed intervention study to compare the transport methods and total trips out of home for all (*n* = 18) participants before vs. after completion of the intervention period, presented in Table 4. Paired *t*-tests demonstrated that a significant reduction in the use of lifts (*p* = 0.014), coupled with non-significant increases in other modes of transport, resulted in an overall reduction in total number of episodes away from home (*p* = 0.031). There was a significant increase in the number of times public transport was used (*p* = 0.035) between pre and post intervention. If modes of transport are combined, i.e., bus, taxi, service, train, courtesy bus (defined as “self- initiated” transport), there was a significant increase between pre and post intervention [M (SD) Pre: 3.55 (5.6), Post 6.05 (5.9) *p* = 0.016].

**Table 4:** Pairwise comparison of before vs. after the intervention period (*n* = 18) M (SD).

**Type of transport**	**Pre**	**Post**	***p*** **-value**
Walking	1.8 (3.5)	1.2 (2.7)	0.389
Bus	2.1 (3.2)	3.1 (4.2)	0.315
Train	0.8 (2.4)	1.3 (2.4)	0.386
Taxi	0.8 (1.8)	1.5 (2.1)	0.142
Service	0.6 (2.5)	0.8 (2.8)	0.104
Lift	4.7 (4.2)	2.8 (3.7)	0.014
Courtesy bus	0 (0)	0.1 (0.2)	0.331
Other	1.7 (3.6)	1.4 (2.0)	0.537
Total	6.7 (3.7)	5.3 (2.0)	0.031

There was no significant change in transport satisfaction across all participants from before [median (IQR) 2 (1–3)] to the time after [Median (IQR) 2 (2–2) *p* = 0.3].

In terms of reporting where the participants went, this is described in Table 5. There were no significant changes in reason participants described they left home. There was a reduction in recreational reasons for leaving the house post intervention, however this was not significant.

**Table 5:** Self-reported episodes away from home M (SD).

	**Education/employment**	**Residential**	**Commercial**	**Health**	**Recreational**	**Social**	**Total**
Baseline	0.4 (0.9)	0.5 (0.7)	1.2 (1.2)	1.2 (1.6)	1.4 (1.9)	1.7 (2.0)	6.4 (3.1)
Post intervention	0.7 (1.2)	0.1 (0.2)	1.1 (1.1)	1.3 (1.6)	0.5 (0.6)	2.3 (1.3)	5.9 (2.7)
Post control	0.4 (0.8)	0.4 (1.0)	1.6 (1.7)	1.5 (1.9)	0.7 (1.1)	1.9 (1.9)	6.4 (3.6)

#### Community Mobility Self-Efficacy Scale

Scores on the Community Mobility Self-Efficacy Scale are described in Table 6. No significant differences were observed between baseline, post intervention, or post control (*p* > 0.05).

**Table 6:** Community mobility self-efficacy scale.

**How confident do you feel about**	**Baseline M (SD)**	**Post intervention** **M (SD)**	**Post control M (SD)**
Being able to stay involved in the community without driving?	6.2 (2.7)	6.7 (2.8)	6.2 (3.2)
Having your health and medical needs met without driving?	7.5 (2.9)	7.6 (3.2)	7.2 (3.2)
About discussing driving and no longer driving with your family and/or health professional?	8.3 (1.8)	6.9 (3.0)	7.3 (3.0)
Finding alternative transport options to get to necessary community activities and appointments?	6.5 (3.0)	6.9 (3.0)	6.9 (3.0)
Staying involved in activities that are important to you without driving?	6.8 (3.0)	6.9 (2.9)	6.6 (3.0)
Staying safe while getting around in the community without driving?	7.1 (2.5)	7.3 (3.2)	7.3 (2.7)
Being able to leave the house without driving?	7.5 (2.6)	7.3 (3.1)	7.2 (3.2)
Being able to stay in contact with friends and family without driving?	6.8 (3.3)	7.2 (3.0)	7.1 (3.4)
Talking about no longer driving with your friends and peers?	8.0 (2.4)	7.7 (2.5)	7.6 (2.6)
Total	63.8 (21.1)	61.7 (24.9)	63.1 (25.4)

#### Health-Related Quality of Life

The summary scores from the quality of life questionnaires are presented in Table 7. There was a large increase in the EQ-5D-5L utility scores between the baseline (0.53) and post control (0.52), and the post intervention period (0.89), however this did not reach statistical significance. There was no evidence of a significant change in the EQ-5D VAS or AQoL-6D additive summary score between the between baseline, after the intervention period, or after the control period.

**Table 7:** Quality of life scores.

	**EQ-5D utility scores** **Mean (SD)**	**EQ-5D (VAS)** **Mean (SD)**	**AQoL-6D** **Mean (SD)**
Baseline	0.53 (0.34)	69.5 (17.1)	66.2 (16.0)
Post intervention	0.89 (1.10)	71.1 (21.5)	63.8 (17.0)
Post control	0.52 (0.49)	70.6 (23.2)	64.2 (15.2)

#### Individual Goals

Participation Goals: Thirty-four individual goals were set by 16 participants, with significant increases in both performance from 3.9 (SD 3.0) before the intervention to 7.0 (SD 2.1), and satisfaction from 4.9 (SD 2.8) to 7.3 (SD 1.8) (both *p* < 0.001). These changes are regarded as clinically significant as they are higher than the 2 points of change defined (46). Four major types of goals were identified: 1. Transport information and experience: these goals related to gaining information, experience and confidence in relation to relevant transport services related to their lives. This was the most common type of goal with 16 being set. An example goal was “To feel more confident using buses for longer trips” (Participant 19). 2. Participation (activities and roles) was a category of goals based on the participation outcomes they wished to achieve through involvement in the program. These included social, leisure, work and feeling busy enough. There were 11 of these goals set. An example goal was, Find out about supported work opportunities” (Participant 12). 3. Emotions and attitudes: These goals focused on emotional responses and personal feelings about themselves and driving cessation. Four goals in this category were identified. An example goal was: “To feel less angry about not being able to drive” (Participant 11); 4. Making a change/contributing was a category of goals related to advocacy and changing the overall situations for others as well as themselves. Three of these goals were set. An example goal was “Having a voice to feedback issues associated with transport use” (Participant 9).

#### Cost and Resource Use

Costs of intervention were calculated in Australian Dollars (AUSD), see Table 8. Where costs occurred as a group cost (e.g., Occupational Therapists time, room bookings) the value per person is calculated on the basis of six people attending a group and 1/6 of the cost allocated to each individual. Where costs occurred per individual (e.g., taxis, printing), the individual cost of these resources per person are presented. Taxi costs are averaged over each individual taxi journey and 12 taxi trips allocated per person. The cost of the CarFreeMe Intervention TI, based on six people in each group, was $2617 AUSD each.

**Table 8:** Costs of CarFreeMe T1 intervention.

**Item**	**Utilization per person**	**Cost per person (AUD)**
OT salary plus on costs $63,010 for 5 groups	1/6 of cost of each group	$2,100.33
OT parking costs × 6 sessions per group	1/6 of cost of each group	$14.00
Guest presenters and lifeflow per group	1/6 of each group	$35.00
Stationary (booklets)	Per person	$57.01
Catering costs × 6 sessions	1/6 of cost of each group	$25.05
Taxi costs for participants	12 journeys per person	$385.98
Room bookings	Free *via* organizations	$0.00
Total per person cost		$2,617.37

#### Carers

Only four participants had people who identified as carers and they did not complete the outcomes, thus this information could not be used in analysis.

#### Participant Satisfaction Survey and Researcher Logs

On the completion of the program participants provided feedback on their experiences. They rated satisfaction with aspects of the program and provided feedback on what was useful and what should change. Feedback from 18 participants was analyzed. Satisfaction was rated highly across content (8.83/10), Presentation (9.23/10) and organization (9.06/10). Open responses to questions about the experience were grouped according to content. Feedback included identifying the most useful aspects of the program, and aspects that could change. Positive aspects were grouped into most useful aspects [social, tailored (personalized), skills, information and experience] and outcomes (attitude, confidence, acceptance, feeling not alone, having more knowledge). Examples of verbatim feedback were: “meeting with other people in similar situations gives us confidence to deal with problems” (Group 2), “the immediate hands on experiences” (Group 6), Things that could change—three major issues were identified: increase length to help with learning; reduce time spent on content I am already familiar with (content was not consistently identified) and consider timing in the rehabilitation process (mainly identifying that they could have benefited from earlier access). Example feedback included “Needs to be a little longer as to become more long term. That is so I can retain in my long term memory” (Group 6).

Researcher logs and reflections indicated that the optimal timing for recruitment was an important consideration, in particular engaging with potential participants within the first 1–2 years following traumatic injury. During this time, people with traumatic injuries were often engaged in other rehabilitation services so their perceived need for the program was reduced. Potential participants were also reluctant to participate in a program outside their existing clinical service where there was established trust and rapport with clinicians. Finally, some participants expressed being unwilling to participate in a community mobility support and education program due to their expectation of being able to return to driving in future.

## Discussion

This study compared the benefits of a 6-week group based support and education program, the CarFreeMe TI, delivered in community settings, to an information sheet of community transport options and was unable to show any difference in community mobility on the primary outcome measure the GPS, however outcome data was incomplete. Despite a standardized process of information provision, regular reminders, the participants had difficulty keeping the devices charged and consistently carrying them when they left the house for their activity over the 7-day data collection period. The QSTARZ BT-Q1000XT is considered the gold standard for research with accuracy within one meter (28), and we have previously utilized to effectively collect full data sets for people following amputations (*n* = 47) (30) and community dwelling older people (*n* = 46) (29) to measure community mobility. Figure 2 shows how the data is recorded and can be presented to show community participation journeys.

Other research by an investigator (JL) has successful recorded outdoor locations using GPS on a smartphone, a passive data collection method with older people with mild cognitive impairment and dementia (31). This approach resulted in participants recording a mean of 161.5/168 h in a week of recording. An accessible, supportive approach was used to support understanding, consent, and practical considerations. Benefits of the approach included being able to monitor whether data were coming in through a data portal, and lower stigma of a mainstream device. As the majority of the participants had a TBI (80%) in the study reported on in this article, there is likely to have been cognitive changes which would have influenced their ability to remember to charge and take the QSTARZ device with them. The method used by Liddle et al. is recommended in future research as this technology reduces burden on participants, compared to self-reported diaries, and may increase accuracy (31) as a complete data set is more likely as a smartphone is generally routinely taken on outings. An approach which combines passively collects data in an accessible way, with self-reported satisfaction and meaning of travel is recommended to enable richer insights.

There was little change in the number or type of visits away from home, after the CarfreeMe T1 intervention, in fact there was a slight reduction. Some potential reasons for this reduction include that since the outcome was taken the week after completing the intervention, participants may have been fatigued after attending a program for 6 weeks, and therefore not venture out as much that week. Also, the nature of changes in transport use that the participants identified in their goals are likely to have slower changes to everyday routines. Participant feedback was that they felt they needed a longer time for intervention, so less intensive support over a longer period of time, with consideration of check-ins or a number of follow up sessions over time to reinforce learnings and problem solve issues identified as confidence with community participation develops, should be considered. This need for repetition and ongoing practice are known clinical strategies for rehabilitation following TBI.

What is not known from the objective GPS data is whether when participants in this study went out, they visited multiple locations and went further distances following the intervention. Other research related to older drivers (18) aimed to increase the trips out of home by a frequency of one, given that when people stop driving, they tend to go out for longer and do multiple activities. Thus, objective measurement of the nature of visit, which are potentially multiple as well of locations, need to be considered in future studies.

From the self-reported data of the participants community mobility, the intervention did not essentially change the patterns (when and where they went), however was effective in changing the mode of transport use, which achieved significance (how they traveled there). At baseline the participants main mode of transport was lifts from others, considered a passive form of transport. At the end of Phase two there was an increased use of public transport, with an average of two episodes per week increase, and self-initiated transport overall. There was also a trend in the reduction of lifts and walking post intervention. Therefore, there was an overall trend of more independence in organizing transport with less reliance on favors or just walking, following the intervention. Immediately post-intervention there was an increased use of services for transport which required the participants to pre organize, demonstrating more independence. This may also be a factor in the reduction of the number of times participants went out following the interventions as it was more effortful, and people may require time to adjust to this within their daily routine. This was found in the study examining the adjustment to loss of driving in TBI, where community participation without driving was complicated because of the difficulties and complexities of examining the use of alternative forms of transport (49).

Within this small sample, there was no change in community self-efficacy. These results are not in concordance with other studies, for example in the context of older drivers where after the intervention of the UQDRIVE (an earlier version of the CarFreeMe TI) (18), aspects of the Community Mobility Self-efficacy Scale demonstrated significant improvements following intervention including: “How confident do you feel about being able to stay involved in the community without driving?”, Item 5: “How confident do you feel about finding alternative transport options to get to necessary community activities and appointments?”, and Item 6: “How confident do you feel about staying involved in activities that are important to you without driving?” (18). In comparison the participants with traumatic injuries in this study were a relatively long time after their injuries (median 6 years) and self-efficacy scores related to community mobility were relatively high at baseline. It could be assumed that due to the length of time since their injury and thus driving cessation they had mostly adapted to the new normal, whereas the older drivers recruited in the Liddle et al. study (18) had stopped driving for any reason and considered driving cessation to be a current issue. Research describing the process of driving cessation for people after TBI identified that the process was very different from the typical experience of older people who stop driving for a variety of reasons. Clinical approaches that consider the timing and processes have been identified as important by health teams working with people after acquired brain injury (17).

Results show an improved self-rated satisfaction and performance in individualized goals related to community mobility and participation in the Phase One intervention group, which was statistically significant. This suggests the CarFreeMe TI intervention, was effective in supporting the personalized goals around community mobility such as confidence in use of public transport, emotions about driving cessation, advocacy related to not driving, and exploring work opportunities. Thus, the CarfreeMe T1 intervention led to an increased perception of goal satisfaction, suggesting that these had not been addressed in other rehabilitation settings, or potentially they had not been willing to accept intervention related to community mobility when hoping to return to driving. The continued meeting of transport and lifestyle goals also suggests that people may need access to therapy in this area for a prolonged period after the traumatic injuries. Additionally, the sharing of experiences, which was embedded throughout the program modules was reported as being highly valued by participants.

This is supported by research with stakeholders in the TBI field (49), which found that supports and clinical processes need to consider multiple factors, including a person's readiness to consider alternatives, formal requirements (legal requirements related to medical fitness to drive, waiting lists for assessments) and participation needs (49). A particularly challenging period during early rehabilitation was noted. It was called the “on hold” period, where a person's main focus is on driving, but they are not able to progress this goal. Not being able to successful navigate this time with the rehabilitation team can lead to distress, disengagement from rehabilitation generally, conflict with family and unsafe driving decisions (unlicensed driving). A need for clinical approaches responsive to the process of driving and driving cessation after traumatic injury is clear, and consideration of both practical and emotional aspects. This was the case for one participant who described as still feeling angry about not driving, when over 2 years after injury—supporting the need for education to focus on the emotion surrounding not driving. It is important that in terms of understanding community mobility and participation that we move beyond assuming more is better, and to also consider perceptions, meaning and satisfaction (50) for each individual. The improvements in participant goal performance and satisfaction illustrate that people are still meeting clinical goals in this stage of their rehabilitation, a long time after the injury has occurred.

The results showed a large increase in mean EQ-5D utility scores between baseline (0.53) and post intervention (0.89, difference 0.36), and post control (0.52) and post intervention (difference of 0.37). Although this did not reach statistical significance, it is three times the minimal clinically important differences reported in stroke patients undergoing rehabilitation (0.1) and larger than those reported for multiple countries using a simulation approach (0.072–0.101) (51, 52). The post-intervention utility score shows a return to health-related quality of life levels similar to the South Australian general population norms, the context of which the study occurred, which includes a large proportion of healthy and young (aged 15 years and over) individuals (0.91) (53). However, we did not find a significant change in our other measures of quality of life. EQ-5D VAS scores did not significantly change. Minimal responsiveness to change for the EQ-5D VAS in people undergoing stroke rehabilitation has previously been reported (46).

The costs of providing the program were $2,617 per person, with the vast majority of those costs in the Occupational Therapists' salary. This program appears relatively low cost, when compared with other rehabilitation interventions aiming to increase community reintegration including those conducted in an in-patient setting, which can cost over €60,000 (24, 25). When compared against a potential to increase the quality of life of the person, the costs of the current program appear worthwhile.

The main limitation of this study is the lack of a complete data set for the primary outcome. The frequency of phone call reminders was based on the clinical recommendation of the blinded assessor (who issued the device) and the participant self-identified preference. For some participants, this was every 1 or 2 days but for other participants it was less regular. For example, if a participant identified having a carer who would monitor use and charging the device, then only one reminder was given. Signs were also used as visual reminders for charging and carrying the GPS (i.e., sign on front door as a prompt before leaving). A standardized approach, such as a daily text message to all participants and follow-up phone calls where indicated, may have resulted in better usage of trackers. We also did not have the option to access GPS data remotely to verify compliance, so this meant researchers relied on participant self-report of compliance with usage and charging. Future studies examining traumatic injuries and community participation should use other community mobility outcomes measures such as GPS location on smartphones to support data collection.

The other limitation is the small sample size and thus results should be interpreted with caution. With the sample size calculation based on a mean difference of one trip out of home, the total number of trips in both intervention and control group were much lower than that reported in the Hordacre et al. (30) study on which the calculations were based, thus it is unlikely that a difference was possible even if the sample size was reached. Potentially the population of people after traumatic injuries may have different patterns of community mobility and a more in-depth understanding of lifespace in detail, may be required prior to establishing power calculations for future trials. Furthermore, the results may not be generalizable as are specific to the participants locations and context of transport and the environment in terms of community mobility.

The number of eligible individuals was not high with the main reason for those who enquired to not being included was a different diagnosis, with stroke being the most common. This suggests that future work should consider the effectiveness of a community group-based education group to improve community participation with stroke survivors. The population of interest, that is people with traumatic injuries, were more challenging to recruit than other studies evaluating a group-based community mobility intervention, in an earlier version of CarFreeMe, the UQDrive, in which a total sample of older drivers of 131 were recruited (18). Recruitment of people following traumatic injuries, was uniquely affected by those earlier on after a TBI still having a goal of return to driving (49) and thus not open to education related to community participation without driving, which was provided in feedback from referral sources to the study. Furthermore, recruitment would have been enhanced if the intervention was embedded in a rehabilitation service, rather than from community sources. The completion of this research has led to a change in practice which although not as comprehensive as the CarFreeMe TI, is a driving cessation clinic implemented in the rehabilitation service for people to be referred when they are do not successfully resume driving to be offered support by an occupational therapist to adjust to not driving and promote community mobility.

Integration of education and support programs like the CarFreeMe TI earlier in rehabilitation to support confidence in community mobility should be considered, as there may be a delay in medical clearance to undergo driving assessment, or the need to wait for recovery and some people will not be capable to return to driving after traumatic injuries. Flexible delivery approaches of the program where it may be available intermittently over a much longer period, spanning from awareness raising and interim experiences with alternative transport while driving is still a goal, to support the transition to participating in the community, and ongoing support as new issues arise, need exploration. This may also reduce the costs of providing the program as would be scaffolded into existing clinical approaches and processes.

Despite these limitations, the study provides the first evidence that community mobility group-based education offers benefits for people with traumatic injuries, and proposes a comprehensive education program for implementation. This program should include individualized goals, with content to include planning and use of alternative transport, advocacy, adjustment to loss and change related to driving. Future research should consider driving and mobility habits prior to traumatic injuries and evaluation over time to see if changes are made and sustained in community mobility after completion of the program. This was beyond the scope of this study, which would have required more resources.

In conclusion, the findings from this study show that the Community Mobility Group Intervention (CarFreeMe TI) is effective in improving mode of transport use and perception of goal performance/satisfaction and quality of life for people with traumatic injuries. Further investigation is required to explore how community mobility intervention can occur earlier in the injury trajectory, with and without driving cessation, and ways to offer the intervention within a rehabilitation pathway/system more gradually over time.

## Data Availability Statement

The raw data supporting the conclusions of this article will be made available by the authors, without undue reservation.

## Ethics Statement

This study was carried out in accordance with Good Clinical Practice (GCP), according to the National Statement on Ethical Conduct in Human Research, and within the laws and regulations of the country in which the research is conducted. All subjects gave written informed consent in accordance with the declaration of Helsinki. The protocol was approved by the Southern Adelaide Clinical Health Research committee (OFR # 42.16 - HREC/16/SAC/47) and published.

## Author Contributions

SG and JL conceived the study and participated in its design/coordination and drafted the manuscript. CB participated in design of the trial process and data analysis. AB and ZA-W participated in oversight of the trial process. AN was involved in the recruitment and data acquisition of all participants and helped draft the manuscript. All authors contributed to the design and conduct of the clinical trial, read, and approved the final manuscript.

## Funding

This study was funded by the Lifetime Support Authority of South Australia.

## Conflict of Interest

JL is a founder of the CarFreeMe intervention. The remaining authors declare that the research was conducted in the absence of any commercial or financial relationships that could be construed as a potential conflict of interest.

## Publisher's Note

All claims expressed in this article are solely those of the authors and do not necessarily represent those of their affiliated organizations, or those of the publisher, the editors and the reviewers. Any product that may be evaluated in this article, or claim that may be made by its manufacturer, is not guaranteed or endorsed by the publisher.
